# Capsaicin 8% patch repeat treatment plus standard of care (SOC) versus SOC alone in painful diabetic peripheral neuropathy: a randomised, 52-week, open-label, safety study

**DOI:** 10.1186/s12883-016-0752-7

**Published:** 2016-12-06

**Authors:** Aaron I. Vinik, Serge Perrot, Etta J. Vinik, Ladislav Pazdera, Hélène Jacobs, Malcolm Stoker, Stephen K. Long, Robert J. Snijder, Marjolijne van der Stoep, Enrique Ortega, Nathaniel Katz

**Affiliations:** 1 Eastern Virginia Medical School, Strelitz Diabetes Center, 855 W Brambleton Avenue, Room 2018, Norfolk, VA 23510 USA; 2 Hôpital Hôtel Dieu, Paris Descartes University, Paris, France; 3 Vestra Clinics - Dedicated Research Clinics, Rychnov nad Kneznou, Czech Republic; 4 Astellas Pharma Europe B. V, Leiden, The Netherlands; 5 Hospital Rio Hortega, Valladolid, Spain; 6 Analgesic Solutions, Natick, MA USA; 7 Tufts University School of Medicine, Boston, MA USA; 8 INC Research, Camberley, UK

**Keywords:** Capsaicin 8% patch, Norfolk QOL-DN, UENS, TPRV1, Painful diabetic peripheral neuropathy

## Abstract

**Background:**

This 52-week study evaluated the long-term safety and tolerability of capsaicin 8% w/w (179 mg) patch repeat treatment plus standard of care (SOC) versus SOC alone in painful diabetic peripheral neuropathy (PDPN).

**Methods:**

Phase 3, multinational, open-label, randomised, controlled, 52-week safety study, conducted in Europe. Patients were randomised to capsaicin 8% patch repeat treatment (30 or 60 min; 1–7 treatments with ≥ 8-week intervals) to painful areas of the feet plus SOC, or SOC alone. The primary objective was the safety of capsaicin 8% patch repeat treatment (30 min and 60 min applications) plus SOC versus SOC alone over 52 weeks, assessed by changes in Norfolk Quality of Life-Diabetic Neuropathy (QOL-DN) total score from baseline to end of study (EOS). Secondary safety endpoints included Utah Early Neuropathy Scale (UENS) assessments and standardised testing of sensory perception and reflex function.

**Results:**

Overall, 468 patients were randomised (30 min plus SOC, *n* = 156; 60 min plus SOC, *n* = 157; SOC alone, *n* = 155). By EoS, mean changes in Norfolk QOL-DN total score from baseline [estimated mean difference versus SOC alone; 90% CI for difference] were: 30 min plus SOC, −27.6% [−20.9; −31.7, −10.1]; 60 min plus SOC, −32.8% [−26.1; −36.8, −15.4]; SOC alone, −6.7%. Mean changes [difference versus SOC alone] in UENS total score by EoS versus baseline were: 30 min plus SOC, −2.1 [−0.9; −1.8, 0.1]; 60 min plus SOC, −3.0 [−1.7; −2.7, −0.8]; SOC alone, −1.2. No detrimental deterioration was observed in any of the Norfolk or UENS subscales by EoS with capsaicin. Also, no worsening in sensory perception testing of sharp, warm, cold and vibration stimuli was found with capsaicin by EoS. Capsaicin treatment was well tolerated and the most frequent treatment-emergent adverse events were application site pain (30 min, 28.2%; 60 min, 29.3%), burning sensation (30 min, 9.0%; 60 min, 9.6%) and application site erythema (30 min, 7.7%; 60 min, 8.9%).

**Conclusion:**

In patients with PDPN, capsaicin 8% patch repeat treatment plus SOC over 52 weeks was well tolerated with no negative functional or neurological effects compared with SOC alone.

**Trial Registration:**

ClinicalTrials.gov registration: NCT01478607. Date of registration November 21, 2011; retrospectively registered.

**Electronic supplementary material:**

The online version of this article (doi:10.1186/s12883-016-0752-7) contains supplementary material, which is available to authorized users.

## Background

Peripheral neuropathic pain (PNP) is widely recognised to have a significant impact on quality of life (QOL) [1]. Painful diabetic peripheral neuropathy (PDPN) has been shown to affect many dimensions of patient QOL, including mood, sleep, work, self-esteem and social relationships, and has a particular impact on individuals with suboptimally managed pain [2, 3]. Approximately one in four people with type 2 diabetes will experience some level of PDPN [4], which often presents as numbness, tingling, burning, aching, electric shocks, or lancinating pains [5].

Many patients with PDPN remain undiagnosed or undertreated and few experience complete resolution of pain. There is a clear unmet need for new therapeutic options to improve current standard of care (SOC); available treatments such as antidepressants, antiepileptic drugs and opioids are often limited by contraindications and safety issues, and frequently have insufficient efficacy to achieve adequate pain relief [6–8]. One alternative to these treatments is the capsaicin 8% patch, which contains 179 mg or 8% weight for weight capsaicin and is optimised for rapid delivery of a high concentration of capsaicin directly into the skin [9]. Defunctionalisation of hyperactive nociceptors in the skin induced by the rapid delivery of capsaicin provides fast, targeted, and sustained pain relief after a single treatment. Furthermore, local application of the capsaicin 8% patch provides minimal systemic absorption, without the potential for drug-drug interactions or requirement for dose adjustment in elderly patients or patients with renal or hepatic impairment [10].

The capsaicin 8% patch is well tolerated and provides effective relief of pain for a variety of types of PNP [11–16]. A single capsaicin 8% patch treatment has demonstrated significant improvements in pain relief versus a placebo patch over 12 weeks, and was well tolerated with no sensory deterioration in patients with PDPN [16]. The present study in patients with PDPN (PACE) was the first evaluation of the long-term safety and tolerability of capsaicin 8% patch repeat treatment plus SOC, compared with SOC alone, over 52 weeks. The study had an open-label design; it primarily assessed the safety of capsaicin 8% patch repeat treatment, with efficacy of the capsaicin 8% patch in PDPN assessed in the double-blind STEP study [16]. In this study, the Norfolk Quality of Life-Diabetic Neuropathy (QOL-DN) questionnaire was chosen as the primary endpoint to assess the safety of capsaicin treatment. The Norfolk scale is a validated patient-reported outcome questionnaire, which captures the entire impact of nerve fibre dysfunction on QOL in diabetic neuropathy [17]. The Norfolk tool includes the concentration of symptoms in the extremities and subtle loss of function, such as fine motor impairments, slight sensory changes, unique problems with proprioception and balance and autonomic symptoms. The Norfolk QOL-DN scale was therefore used to assess any functional consequences associated with potentially deleterious effects of capsaicin treatment on peripheral nerve endings in patients with PDPN.

## Methods

### Study design

PACE was a Phase 3, multinational, open-label, randomised, controlled, 52-week safety study, conducted in Europe between November 2011 and February 2014 (ClinicalTrials.gov Identifier: NCT01478607). The primary objective assessed the safety of repeat treatment with the capsaicin 8% patch (QUTENZA^TM^ 179 mg capsaicin patch, obtained from Astellas Pharma Europe B.V., Leiden, The Netherlands) in patients with PDPN.

**Fig. 1:**
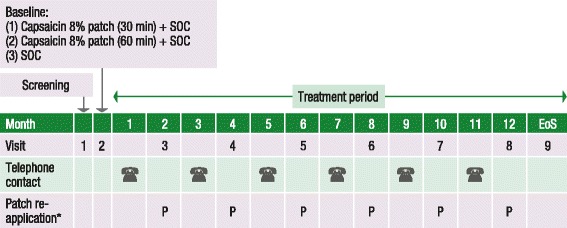
Study design *Capsaicin 8% patch treatment (Groups 1 and 2) took place at scheduled bi-monthly visits (P) or unscheduled visit at intervals of at least 8 weeks. EoS visit for Groups 1 and 2 took place between 8 and 12 weeks after last patch application if patch was applied at Visit 8 (Month 12) and between Week 52 and 56 for patients without a patch application at Visit 8 (Month 12). EoS visit for Group 3 took place between Week 52 and 56. *EoS* end of study, *SOC* standard of care, *UENS* Utah Early Neuropathy Scale

Following a screening visit, patients were assigned a six-digit subject number allocated sequentially according to site and randomised to capsaicin 8% patch (30 min) plus SOC or capsaicin 8% patch (60 min) plus SOC or SOC alone in a ratio of 1:1:1 by chronological order of enrolment to receive treatment with the capsaicin 8% patch to painful areas of the feet for either 30 min plus SOC, 60 min plus SOC, or SOC alone. All patients were pretreated with a eutectic mixture of local anaesthetics (EMLA) containing lidocaine 2.5% and prilocaine 2.5%, to limit pain or discomfort during the application period. The 30-min application time was chosen to align with the approved Summary of Product Characteristics [18]. A 60-min application time was also evaluated in order to ensure that the safety objectives of the study were fully covered with respect to possible exposure periods. SOC was optimised for each patient at the discretion of each investigator and was assessed at clinic visits and on days 1 to 5 post-treatment, by completion of a rescue pain medication diary. The treatment area was mapped at screening and baseline visits, and re-mapped before treatment if the treatment area changed. Treatment borders were defined by the most painful areas of the feet, up to a total combined surface area of 1120 cm^2^ (four patches) for both feet. Assessments were scheduled every two months; clinic visits were scheduled for Month 2, 4, 6, 8, 10, and 12, and telephone contact was scheduled at Month 1, 3, 5, 7, 9, and 11. Capsaicin 8% patch retreatment could occur at both scheduled and unscheduled clinic visits at the investigator’s discretion, but only after at least 8 weeks had elapsed since the last treatment (Fig. 1).

Patients could not receive more than seven capsaicin 8% patch treatments during the study. Although patients and investigators were unblinded throughout the study, physicians assessing neurological function were blinded to treatment and not involved in the study in any other manner.

### Patients

Patients were aged ≥18 years with a diagnosis of PDPN due to type 1 or type 2 diabetes mellitus for ≥1 year prior to the screening visit. Key criteria for inclusion and exclusion are presented in Table 1.

**Table 1:** Key inclusion and exclusion criteria

Inclusion criteria	
• Aged ≥ 18 years with a diagnosis of PDPN confirmed by a score ≥ 3 on the MNSI • HbA1c ≤ 9% (74.9 mmol/mol) at 3–6 months prior to screening and at screening	• Stable glycaemic control for ≥ 6 months prior to screening visit • Average daily pain score over the last 24 h ≥ 4 (question 5 of BPI-DN) at the screening and the baseline visit
Exclusion criteria	
• Primary pain associated with PDPN in the ankles or above • Significant pain (moderate or above) due to an aetiology other than PDPN • Any amputation of lower extremity • Clinically significant cardiovascular disease within 6 months prior to screening visit • Any active signs of skin inflammation around onychomycosis sites such as tenderness, redness, swelling or drainage • Body mass index ≥ 40 kg/m^2^ • Hypersensitivity to capsaicin any capsaicin 8% patch excipients, EMLA ingredients, or adhesives • Use of oral or transdermal opioids within 7 days preceding patch application at baseline	• Pain that could not be clearly differentiated from, or conditions that might have interfered with, the assessment of PDPN, e.g., claudication, fasciitis tendinitis and arthritis • Current or previous foot ulcer • Severe renal disease as defined by a creatinine clearance < 30 mL/min • Significant peripheral vascular disease^a^ • Impaired glucose tolerance only – without diabetes mellitus • Previous treatment with capsaicin 8% patch • Use of any topical pain medication on the painful areas within 7 days preceding patch application at baseline

*BPI-DN* Brief pain inventory-diabetic neuropathy version, *EMLA* eutectic mixture of local anaesthetics, *HbA1c* glycosylated haemoglobin of A1c, *MNSI* Michigan neuropathy screening instrument, *PDPN* painful diabetic peripheral neuropathy

^a^Intermittent claudication or lack of pulsation of either the dorsal pedis of posterior tibias artery, or ankle-brachial systolic BP index of 0.80

## Safety endpoints

### Primary endpoint

#### Norfolk QOL-DN Scale

The primary objective was to evaluate the safety of repeat treatment with the capsaicin 8% patch, assessed by the percentage change from baseline to end of study (EoS) in the Norfolk QOL-DN total score. The scale has been shown to correlate with clinical Total Neuropathy Score along with the different features of diabetic neuropathy such as small fibre function (including loss of pain and thermal sensation), large fibre function (including motor function and touch/pressure discrimination) and autonomic nerve function [19]. The Norfolk QOL-DN scale was specifically developed to reliably measure changes in nerve function that translate into changes in QOL, activities of daily living, and health of the individual, where a reduction in score is associated with improved function [17]. In this study, it was used to assess any functional consequences of potential small nerve fibre dysfunction that may have been associated with capsaicin 8% patch repeat treatment and adversely affected QOL.

#### Secondary endpoints

Secondary safety variables evaluated to support the primary endpoint included Norfolk QOL-DN subscale scores, Utah Early Neuropathy Scale (UENS) [20] assessments and standardised testing of sensory perception and reflex function. Average pain score, pain severity index, pain interference index, obtained from the Brief Pain Inventory-Diabetic Neuropathy (question 5), response rates and Patient Global Impression of Change were recorded as other secondary endpoints in this study and will be reported separately.

#### Secondary Norfolk QOL-DN endpoints

Secondary safety variables related to Norfolk QOL-DN included: percentage change from baseline in Norfolk QOL-DN total score by number of capsaicin treatments; percentage change from baseline in Norfolk QOL-DN subscale scores (‘small fibre’, ‘symptoms’, ‘autonomic’, ‘physical functioning’, ‘activities of daily living’); and absolute Norfolk QOL-DN total score. A reduction in subscale score was associated with improved function.

#### Utah early neuropathy scale

The UENS, a validated clinical tool developed to detect and quantify signs of early neuropathy and identify modest changes in the severity and spatial distribution of sensation, was used to assess any functional consequences of capsaicin treatment. A reduction in score indicates improvement in sensory function over time. Endpoints related to the UENS included: change from baseline in UENS total score; change from baseline in UENS total score by number of capsaicin treatments; change from baseline in UENS subscale scores (‘pinprick sensation’, ‘motor’, ‘allodynia/hyperaesthesia’, ‘large fibre’, ‘deep tendon reflex’); clinically significant change in UENS total score (defined as a decrease of > 4 points from baseline); and absolute UENS total score.

#### Sensory perception, reflex function and tolerability

**Fig. 2:**
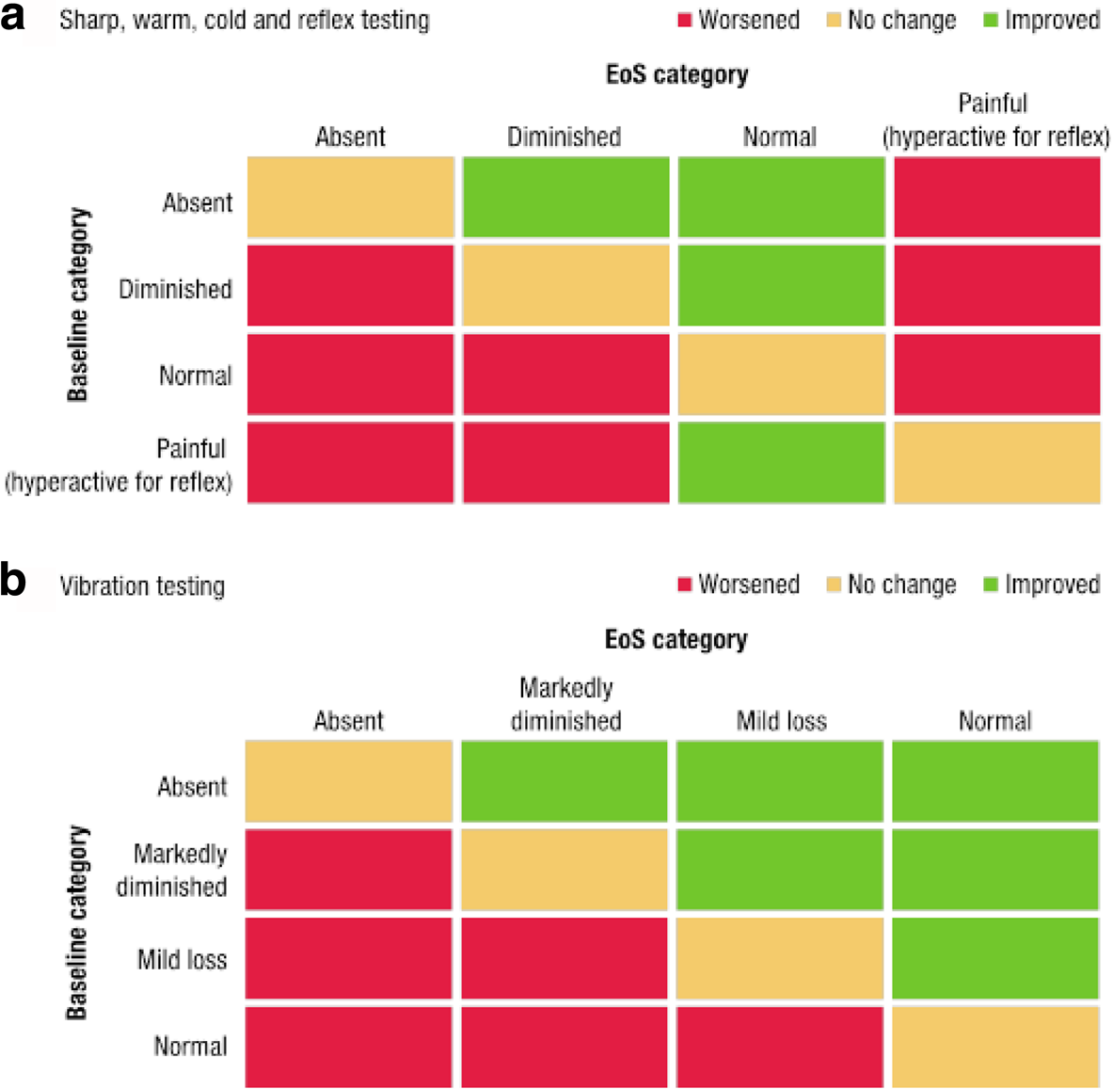
Category shift schema from baseline to EoS. *EoS* end of study

Sensory examination was performed by neurologists as well as by non-neurologist physician-investigators given study training, and were ideally performed by the same person (Additional file 1). Physicians conducting sensory examinations were blinded to treatment. ‘Bedside’ sensory and reflex testing was performed on both feet at baseline and EoS to identify any clinically relevant deficits in sensory function. Ratings of evoked sensation were compared with an asymptomatic site and recorded using standardised categorical reporting scales: assessment of warm, cold, and sharp sensations were rated as ‘absent’, ‘diminished’, ‘normal’, or ‘painful’. Sensation of vibration on the dorsal surface of the great toe was rated as ‘absent’, ‘markedly diminished’, ‘mild loss’, or ‘normal’ sensation. Testing areas on the dorsal surface included the great toe, midpoint and medial malleolus and on the plantar surface included the ball and midpoint. Achilles tendon reflex assessment was rated as ‘absent’, ‘diminished’, ‘normal’, ‘hyperactive’, or ‘clonus’. To assess the proportion of patients with changes in sensory perception or reflex category, patients were judged to have improved, stayed the same, or worsened depending on the change in reported category at EoS versus baseline (Fig. 2).

A post-hoc analysis in patients who received the maximum of seven capsaicin 8% patch treatments was performed to determine within-group changes of sensory perception testing over time.

Adverse events (AEs) observed after randomisation (post-randomisation AEs [PRAEs]) were collected for all groups, allowing for comparison of the capsaicin 8% patch arms with the SOC arm. Treatment-emergent AEs (TEAEs) were observed following administration of the capsaicin 8% patch, therefore no TEAEs are reported for the SOC alone group. ‘Pain now’ Numeric Pain Rating Scale (NPRS) scores, within 15 min and after 60 min following patch removal, were also assessed during the study.

The change in use of concomitant medication was assessed throughout the study; classes of interest were antidepressants, antiepileptic drugs, and opioids.

### Statistical methods

Regarding sample size, the number of patients planned for the trial were such that safety concerns related to the Norfolk questionnaire would have been picked up. The power calculation for this study used the principle of a non-inferiority study with 90% power and a 95% one sided confidence interval [CI]. The non-inferiority limit was 20%. A clinically meaningful difference in the percentage reduction from baseline on the Norfolk scale was chosen using data from a clinical trial in diabetic peripheral neuropathy with ruboxistaurin [21], which showed a drug effect of 37.2%, and selecting 20% as the lower margin for clinically meaningful effect. A number of statistical measures were used to describe the data: descriptive statistics for absolute and change from baseline values at each visit and at EoS; 90 or 95% CI for the estimated mean difference between each of the active treatment groups against SOC control for change from baseline; percentage change from baseline at each post-baseline analysis visit and EoS. As the objective of the trial was to assess the safety and tolerability of long-term capsaicin 8% patch treatment, no formal statistical testing was performed to calculate *p*-values for the difference between both capsaicin groups and SOC alone. At EoS, for each subject, the last available observation was used with the last observation carried forward (LOCF) imputation method. The results were also analysed using the baseline observation carried forward method.

The safety analysis set (SAS) included all patients who received study treatment and was used for all analyses of safety and analgesic effectiveness.

## Results

### Patient disposition

**Fig. 3:**
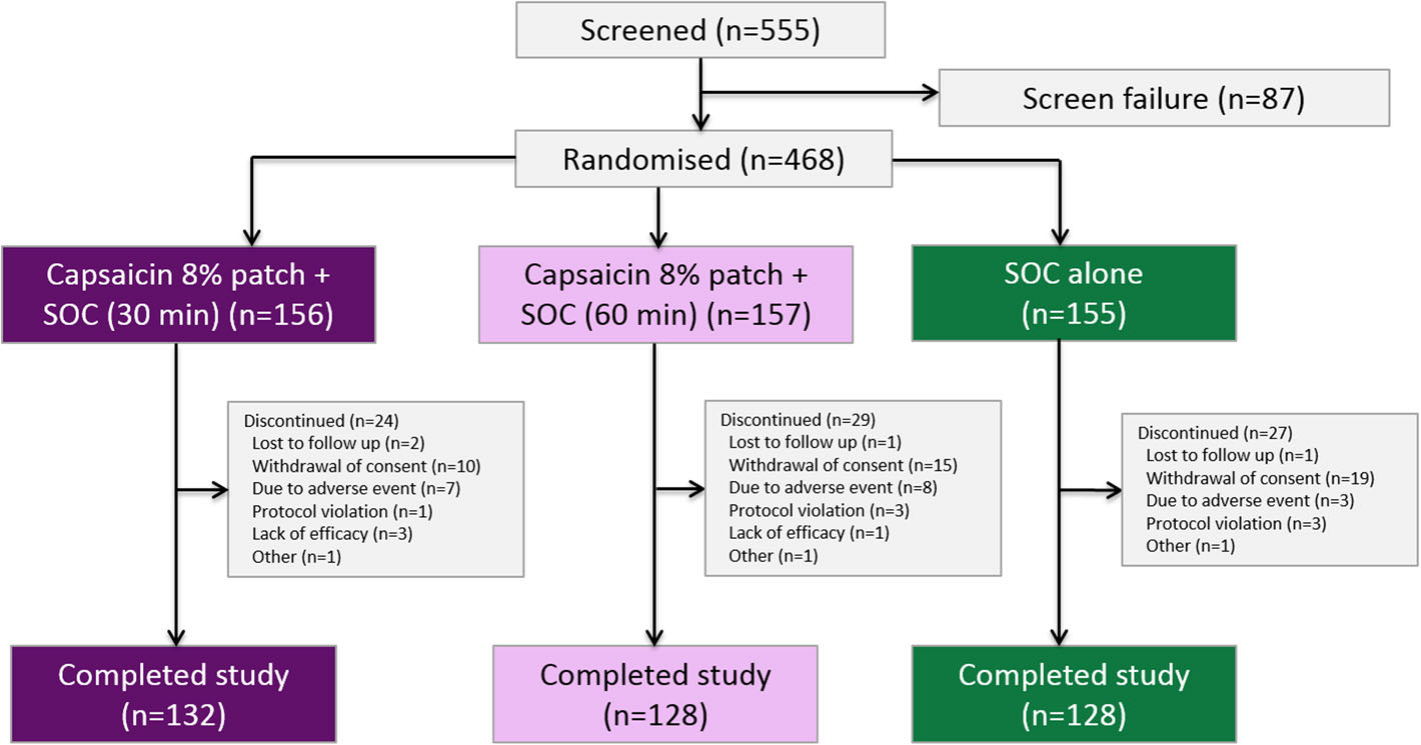
Patient flow. *AE* adverse event, *SOC* standard of care

Of the 555 patients screened, a total of 468 patients were randomised at 71 centres across 11 European countries (30 min plus SOC, *n* = 156; 60 min plus SOC, *n* = 157; SOC alone, *n* = 155). A total of 388 patients completed the study (30 min plus SOC, *n* = 132; 60 min plus SOC, *n* = 128; SOC alone, *n* = 128); 80 patients (17.1%) discontinued the study post baseline, most commonly due to withdrawal of consent (*n* = 44) and adverse events (*n* = 18; Fig. 3).

Baseline characteristics were similar across treatment groups and specifically, were comparable for age, glycosylated haemoglobin of A1c (HbA1c), average daily pain, Norfolk QOL-DN total score, UENS total score, duration of PDPN and use of prior treatments for PDPN (including pain medications and SOC) (Table 2).

**Table 2:** Summary of demographics and baseline characteristics (safety analysis set)

Parameter	Capsaicin 8% patch (30 min) + SOC (*n* = 156)	Capsaicin 8% patch (60 min) + SOC (*n* = 157)	SOC (*n* = 155)
Sex, *n* (%)			
Male	74 (47.4)	79 (50.3)	71 (45.8)
Female	82 (52.6)	78 (49.7)	84 (54.2)
Ethnicity, *n* (%)			
Caucasian	154 (98.7)	155 (98.7)	154 (99.4)
Other	2 (1.3)	2 (1.3)	1 (0.6)
Age, years			
Mean [SD]	60.9 [10.9]	61.0 [10.3]	59.1 [10.3]
Weight, kg			
Mean [SD]	86.6 [14.5]	86.7 [16.4]	89.6 [17.6]
Height, cm			
Mean [SD]	169.7 [8.9]	169.7 [9.0]	169.3 [10.9]
Body mass index, kg/m^2^			
Mean [SD]	30.1 [4.6]	30.1 [5.0]	31.2 [5.0]
Duration of PDPN, years			
Mean [SD]	4.1 [3.7]	4.4 [3.9]	4.4 [3.6]
Pain medications before baseline, *n* (%)			
Overall	70 (44.9)	71 (45.2)	79 (51.0)
Analgesics^a^	56 (35.9)	54 (34.4)	59 (38.1)
Antiepileptics	44 (28.2)	49 (31.2)	52 (33.5)
Psycholeptics	22 (14.1)	19 (12.1)	24 (15.5)
Anti-inflammatory/antirheumatic products	14 (9.0)	12 (7.6)	17 (11.0)
Topical joint/muscular pain products^b^	14 (9.0)	11 (7.0)	15 (9.7)
Baseline average pain score (BPI-DN question 5)			
Mean [SD]	5.6 [1.3]	5.6 [1.4]	5.5 [1.3]
Baseline Norfolk QOL-DN score			
Mean [SD]	42.8 [19.5]	40.6 [18.3]	41.0 [18.5]
Baseline UENS total score			
Mean [SD]	17.0 [7.4]	16.5 [7.0]	15.6 [6.2]
HbA1c at screening			
Mean, % [SD]	7.3 [1.0]	7.4 [1.0]	7.4 [1.0]
Mean, mmol/mol [SD]	56.6 [10.8]	57.5 [10.8]	57.6 [11.4]

*BPI-DN* Brief pain inventory diabetic neuropathy, *HbA1c* glycosylated haemoglobin of A1c, *PDPN* painful diabetic peripheral neuropathy, *QOL-DN* Quality-of-life questionnaire for diabetic neuropathy, *SD* standard deviation, *SOC* standard of care

^a^Analgesics were categorised by analgesics, anilides, natural opium alkaloids, other analgesics and antipyretics, other opioids, pyrazolones and salicylic acid and derivatives

^b^Anti-inflammatory preparations, non-steroidals for topical use, preparations with salicylic acid derivatives

The most commonly prescribed categories of pain medications during the study were analgesics and antiepileptics; the most commonly prescribed drugs for pain during the study were gabapentin and pregabalin (Table 3).

**Table 3:** Pain medication during the study (safety analysis set)

Pain medication^a^	Capsaicin 8% patch (30 min) + SOC (*n* = 156)	Capsaicin 8% patch (60 min) + SOC (*n* = 157)	SOC (*n* = 155)
Overall, *n* (%)	98 (62.8)	105 (66.9)	107 (69.0)
Most commonly used category (>10 % patients in either group), *n* (%)			
Analgesics^b^	79 (50.6)	84 (53.5)	81 (52.3)
Antiepileptics	54 (34.6)	57 (36.3)	73 (47.1)
Topical products for joint and muscular pain	30 (19.2)	35 (22.3)	29 (18.1)
Anti-inflammatory/antirheumatic products	29 (18.6)	35 (22.3)	30 (19.4)
Psycholeptics	24 (15.4)	22 (14.0)	40 (25.8)
Stomatological preparations	18 (11.5)	22 (14.0)	18 (11.6)
Psychoanaleptics	16 (10.3)	6 (3.8)	21 (13.5)
Ophthalmologicals^c^	15 (9.6)	20 (12.7)	16 (10.3)
Most commonly used drugs (>5% patients in any group), *n* (%)			
Gabapentin	26 (16.7)	26 (16.6)	35 (22.6)
Pregabalin	24 (15.4)	22 (14.0)	39 (25.2)
Paracetamol	23 (14.7)	36 (22.9)	6 (3.9)
Tramadol	16 (10.3)	14 (8.9)	6 (3.9)
Diclofenac	12 (7.7)	13 (8.3)	12 (7.7)
Ibuprofen	11 (7.1)	15 (9.6)	14 (9.0)
Metamizole	10 (6.4)	10 (6.4)	5 (3.2)
Duloxetine	9 (5.8)	3 (1.9)	10 (6.5)
Carbamazepine	7 (4.5)	14 (8.9)	10 (6.5)
Alpha lipoic acid	3 (1.9)	1 (0.6)	8 (5.2)

*SOC* standard of care

^a^Medication used for pain (check box of ‘pain medication’ is YES on electronic case report form [eCRF])

^b^Analgesics were categorised by class: anilides, natural opium alkaloids, other analgesics and antipyretics, other opioids, pyrazolones, and salicylic acid and derivatives

^c^Ophthalmologicals (eye treatments) were categorised by anti-inflammatory agents and nonsteroids, local anaesthetics, corticosteroids (plain) and other ophthalmologicals

The average interval between each capsaicin retreatment was 68.4 days in the capsaicin 30-min group and 68.3 days in the 60-min group. The mean number of patches used was similar between capsaicin groups (30 min, 1.53; 60 min, 1.42) and the mean duration of patch application was 30.2 min in the 30-min group and 60.2 min in the 60-min group. Over half of patients in the capsaicin 8% patch groups received the maximum seven capsaicin treatments (167/313 [53.4%]) (Additional file 2: Figure S1 and Additional file 3: Figure S2).

### Safety

#### Norfolk QOL-DN

**Fig. 4:**
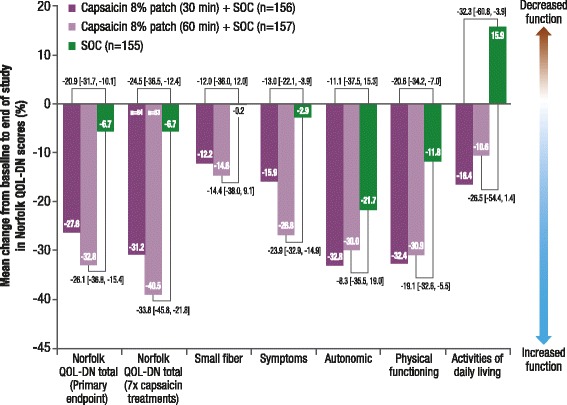
Mean percentage change from baseline to end of study in Norfolk QOL-DN scores (LOCF) (SAS) Treatment group comparisons are least squares mean difference [90% CI]. *CI* confidence interval, *LOCF* last observation carried forward, *SAS* safety analysis set, *SOC* standard of care

No deterioration (denoted by a reduction in score) in mean (estimated mean difference versus SOC alone; 90% CI for difference) Norfolk QOL-DN total score from baseline to EoS was observed in the capsaicin 8% patch plus SOC groups (30 min, −27.6% [−20.9; −31.7, −10.1]; 60 min, −32.8% [−26.1; −36.8, −15.4]) compared with SOC alone (−6.7%) (Fig. 4). By EoS, patients who received the maximum seven capsaicin treatments plus SOC also had no deterioration in Norfolk QOL-DN when compared with the overall SAS population (Fig. 4).

**Fig. 5:**
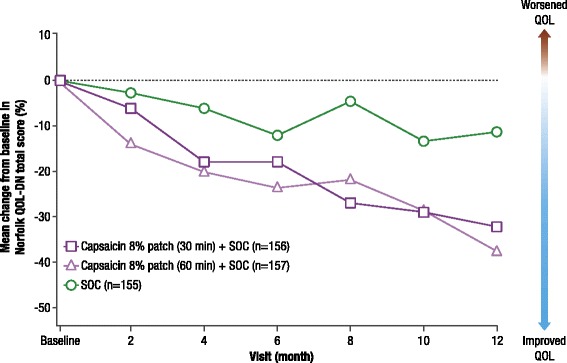
Mean percentage change in Norfolk QOL-DN total score from baseline during the study (SAS) In patients who received a capsaicin treatment at Month 12 and had an end of study visit at Month 14, mean [SD] change in Norfolk total score by Month 14 was: 30 min, −36.1% [51.6] (*n* = 79); 60 min, −40.2% [39.4] (*n* = 76). *SAS* safety analysis set, *SOC* standard of care

The mean difference [90% CI] from baseline in Norfolk QOL-DN total score between groups increased throughout the study (Fig. 5).

In addition, no deterioration in any of the Norfolk QOL-DN subscale scores from baseline to EoS was observed in both capsaicin plus SOC groups versus SOC alone (Fig. 4).

#### Utah early neuropathy scale

**Fig. 6:**
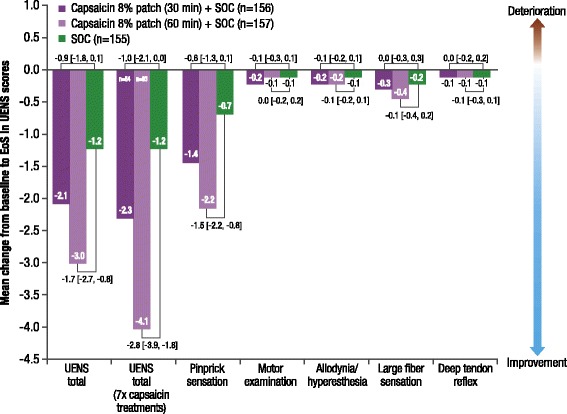
Mean change in UENS total and subscale scores from baseline to EoS (LOCF) (SAS) Treatment group comparisons are least squares mean difference [90% CI]. *CI* confidence interval, *EoS* end of study, *LOCF* last observation carried forward, *SAS* safety analysis set, *SOC* standard of care

The mean change [SD] from baseline to EoS in absolute UENS total score was −2.1 [5.0] with capsaicin 30 min and −3.0 [5.1] with capsaicin 60 min, compared with −1.2 [4.2] in SOC alone group (least squares mean difference [90% CI]: 30 min, −0.9 [−1.8, 0.1]; 60 min: −1.7 [−2.7, −0.8]) (Fig. 6).

No deterioration in UENS total score was noted in patients who received the maximum seven capsaicin treatments plus SOC compared with the overall SAS population (Fig. 6). A clinically significant improvement in UENS total score (defined as a decrease of > 4 points from baseline) was observed in 35.6 and 37.9% of patients in the capsaicin 30-min and capsaicin 60-min groups, respectively, compared with 22.5% of patients in the SOC alone group.

Regarding UENS subscales, no deterioration in mean [SD] ‘pinprick sensation’ score was observed in the capsaicin plus SOC groups compared with SOC alone (30 min, −1.4 [3.84]; 60 min, −2.2 [3.99]; SOC, −0.7 [3.14]). There were no noticeable differences or only minimal changes across treatment groups in the other UENS subscale scores (Fig. 6).

No differences were observed between treatment groups (data not show) using the baseline observation carried forward method.

#### Sensory perception and reflex testing

**Fig. 7:**
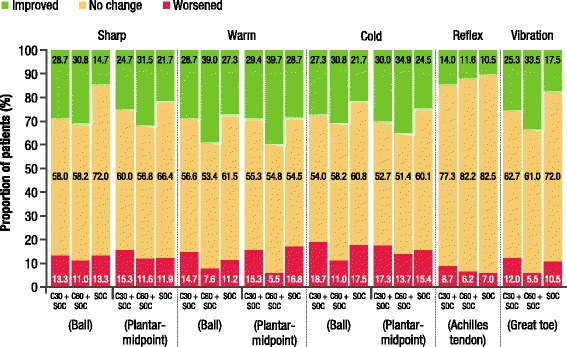
Proportion of patients reporting improved, unchanged, or worsened sensory or reflex function by EoS (SAS) C30 + SOC, capsaicin 8% patch (30 min) + SOC (*n* = 150); C60 + SOC, capsaicin 8% patch (60 min) + SOC (*n* = 146) *EoS* end of study, *SAS* safety analysis set, *SOC* standard of care (*n* = 143); *n* is number of patients with non-missing data

The capsaicin 8% patch had no negative impact on sensory perception and reflex testing and the majority of patients had no change from baseline to EoS (Fig. 7, Additional file 4: Table S1). In patients who received the maximum seven capsaicin treatments, there was also no negative impact from baseline to EoS (Additional file 5: Figure S3). A shift to worsened sensation was reported by a lower proportion of patients in both the overall population and the capsaicin 8% patch seven treatment cohort (Fig. 7, Additional file 5: Figure S3).

**Fig. 8:**
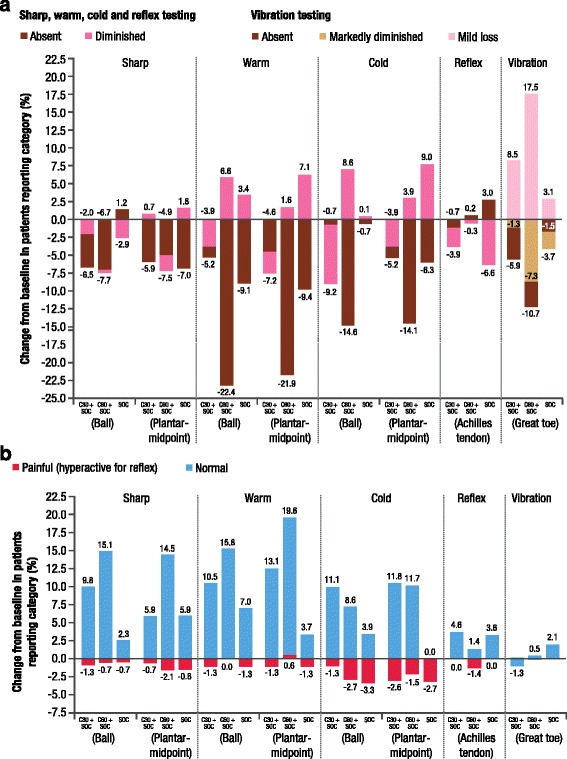
**a** and **b** Change in proportion of patients reporting sensory and reflex testing from baseline to EoS (SAS) C30 + SOC, capsaicin 8% patch (30 min) + SOC (*n* = 156); C60 + SOC, capsaicin 8% patch (60 min) + SOC (*n* = 157) *EoS* end of study, *SAS* safety analysis set, *SOC* standard of care (*n* = 155)

By EoS, the proportion of patients reporting ‘normal’ sensation increased for the majority of tests in all three groups; however, reporting of ‘normal’ sharp (on ball of foot), warm, and cold sensation was greater for capsaicin groups plus SOC versus SOC alone (Fig. 8a). The proportion of patients reporting ‘absent’ sharp (on plantar midpoint), warm, cold and vibration sensation decreased in all groups by EoS, with the greatest change in warm, cold and vibration seen in the capsaicin 60 min plus SOC group (Fig. 8b). There was also a decrease in the proportion of patients, in all groups, reporting ‘absent’ or ‘markedly diminished’ vibration sensation by EoS, and this was accompanied by a corresponding increase in ‘mild loss’ vibration in all groups (Fig. 8b). These findings were mirrored in the subset of patients who received the maximum seven capsaicin treatments plus SOC, with more patients in the capsaicin groups reporting ‘normal’ sensation for sharp, warm, and cold tests compared with the SOC alone group (Additional file 6: Figure S4, Additional file 7: Table S2). Also in the seven treatments subset, the proportion of patients reporting ‘absent’ sharp (on plantar midpoint), warm, cold and vibration sensation decreased in all groups by EoS, with the greatest change in warm and cold seen in the capsaicin 60 min plus SOC group (Additional file 8: Figure S5). In addition, fewer patients in the seven treatment subset reported ‘absent’ vibration sensation by EoS, accompanied by a corresponding increase in ‘mild loss’ vibration (Additional file 8: Figure S5).

#### Tolerability

Mean ‘pain now’ NPRS scores after patch application were low (≤3.5); peak mean pain scores were observed within 15 min after the first patch removal (3.5; median 3.0, interquartile range 2.0–5.0) to 60 min after the first patch removal (3.3; median 3.0, interquartile range 1.0–5.0). However, ‘pain now’ NPRS scores decreased after the second patch removal and were stable from the fifth to the last patch removal. More patients in the capsaicin 8% patch 60-min group used concomitant analgesics for application site-related pain (29.9%) compared with the 30-min group (22.4%).

The proportion of patients reporting any PRAE was 67.3% (30 min plus SOC), 69.4% (60 min plus SOC) and 48.4% (SOC alone) (Table 4), and the majority of PRAEs were of mild or moderate severity.

**Table 4:** Summary of PRAEs, TEAEs, and drug-related TEAEs (safety analysis set)

Event, *n* (%)	Capsaicin 8% patch (30 min) + SOC (*n* = 156)	Capsaicin 8% patch (60 min) + SOC (*n* = 157)	SOC (*n* = 155)
PRAEs	105 (67.3)	109 (69.4)	75 (48.4)
Mild PRAEs	83 (53.2)	89 (56.7)	55 (35.5)
Moderate PRAEs	50 (32.1)	54 (34.4)	34 (21.9)
Severe PRAEs	19 (12.2)	12 (7.6)	10 (6.5)
PRAEs identified as general disorders or administration site conditions	54 (34.6)	53 (33.8)	10 (6.5)
Application site pain	44 (28.2)	46 (29.3)	0 (0)
Mild	23 (14.7)	28 (17.8)	0 (0)
Moderate	19 (12.2)	16 (10.2)	0 (0)
Severe	2 (1.3)	2 (1.3)	0 (0)
Application site erythema	12 (7.7)	14 (8.9)	0 (0)
Mild	12 (7.7)	13 (8.3)	0 (0)
Moderate	0 (0)	1 (0.6)	0 (0)
Severe	0 (0)	0 (0)	0 (0)
PRAEs leading to permanent discontinuation	7 (4.5)	8 (5.1)	3 (1.9)
TEAEs	104 (66.7)	106 (67.5)	N/A
Application site reactions	60 (38.5)	69 (43.9)	
TEAEs most commonly reported (>5% of each group)			N/A
Application site pain	44 (28.2)	46 (29.3)	
Burning sensation	14 (9.0)	15 (9.6)	
Application site erythema	12 (7.7)	14 (8.9)	
Pain in extremity	6 (3.8)	13 (8.3)	
TEAEs leading to permanent discontinuation	7 (4.5)	8 (5.1)	N/A
Drug-related^a^ TEAEs	62 (39.7)	71 (45.2)	N/A
Drug-related^a^ TEAEs leading to permanent discontinuation	0 (0)	4 (2.5)	N/A
Muscle spasms^b^	0 (0)	1 (0.6)	
Rectal adenocarcinoma^c^	0 (0)	1 (0.6)	
Neuralgia^d^	0 (0)	1 (0.6)	
Plantar psoriasis^e^	0 (0)	1 (0.6)	
Severe TEAEs	19 (12.2)	12 (7.6)	N/A
Drug-related^a^ severe TEAEs	4 (2.6)	3 (1.9)	N/A
Application site pain	2 (1.3)	2 (1.3)	
Rectal adenocarcinoma^c^	0 (0)	1 (0.6)	
Burning sensation	1 (0.6)	0 (0)	
Hypoaesthesia	1 (0.6)	0 (0)	
Serious TEAEs	20 (12.8)	13 (8.3)	N/A
Drug-related^a^ serious TEAEs	0 (0)	2 (1.3)	N/A
Angina pectoris^f^	0 (0)	1 (0.6)	
Accelerated hypertension^g^	0 (0)	1 (0.6)	
Rectal adenocarcinoma^c^	0 (0)	1 (0.6)	
Deaths	1 (0.6)	1 (0.6)	2 (1.3)

*N/A* not applicable in SOC alone group, *PRAE* post-randomisation adverse event, *SOC* standard of care, *TEAE* treatment-emergent adverse event

^a^Possible or probable, as assessed by the investigator, or records where relationship is missing

^b^Muscle spasms in both legs of one patient were considered unlikely to have been caused by the capsaicin 8% patch, but a causal association could not be excluded. The same patient also previously reported cramps in the toes

^c^Rectal adenocarcinoma was considered unlikely to have started during the study and reach grade T3 within 129 days; however, a causal association with the capsaicin 8% patch could not be excluded

^d^One case of neuralgia was considered probably related to the study drug in view of the close temporal association with dosing and the known ability of the capsaicin 8% patch to cause application site pain

^e^Although the capsaicin 8% patch could not be excluded as a cause of one event of plantar psoriasis, the mechanism by which it could cause an autoimmune condition such as psoriasis is unclear and a causal association was considered unlikely

^f, g^Angina pectoris and accelerated hypertension were likely related to the patient’s co-existing ischemic heart disease and hypertension but a causal association with the capsaicin 8% patch could not be excluded

Severe PRAEs were reported by 12.2, 7.6 and 6.5% in the patients in the capsaicin 30 min, capsaicin 60 min, and SOC alone, groups, respectively; overall, these severe PRAEs were most commonly categorised as cardiac disorders, infections and infestations, and musculoskeletal and connective tissue disorders. The difference in PRAEs between the capsaicin plus SOC groups and SOC alone was primarily due to the reporting of application site pain or erythema in the capsaicin groups, which were predominately mild or moderate in severity (Table 4).

TEAEs were similar in the capsaicin 8% patch 30-min and 60-min plus SOC treatment groups (66.7 and 67.5%, respectively) and were generally mild or moderate in severity (Table 4). Over one-third of TEAEs in the capsaicin groups were application site reactions; the type and frequency of these reactions were comparable between both capsaicin groups. The proportion of patients with application site reactions decreased throughout the study, from between the first and second to between the sixth and seventh treatments (application site pain: 30 min, −6.9%; 60 min, −7.9%, burning sensation: 30 min, −2.8%; 60 min −2.2%). Four patients in the 60-min group discontinued due to drug-related TEAEs (four events: muscle spasms, rectal adenocarcinoma, neuralgia, and psoriasis); no drug-related discontinuations were reported in the 30-min group. Severe TEAEs were reported by 12.2% in the 30-min group and 7.6% in the 60-min group; severe TEAEs considered as drug related were reported by 2.6% in the 30-min group and 1.9% in the 60-min group. Serious TEAEs considered as drug related were reported by two patients in the 60-min group (three events: angina pectoris, rectal adenocarcinoma and accelerated hypertension), and by no patients in the 30-min group. These events were officially classified as drug-related by the investigator because causation could not be excluded; however, the sponsor considered it unlikely that repeat treatment with capsaicin 8% patch was the cause of these events. Four deaths were reported during the study: capsaicin 30 min, multiple injuries and brain death (*n* = 1); capsaicin 60 min, hypotension (*n* = 1); SOC alone, pneumonia (*n* = 1), atrial fibrillation (*n* = 1). None of the deaths were considered to be drug related by the investigator. The only finding of note from vital sign and laboratory analyses was that the change in HbA1c from screening to EoS was marginally greater in the SOC alone arm (0.24%), compared with the capsaicin 30 min (0.06%) and 60 min (0.06%) arms, although HbA1c levels were generally controlled throughout the study.

#### Concomitant medications

Overall, there was no decrease in the use of concomitant medications in any of the treatment groups throughout the study; use of concomitant medications was comparable from baseline to EoS in both capsaicin groups (Additional file 9: Table S3). The proportion of patients using antiepileptics at the end of the study was comparable with the proportion reported at baseline for both capsaicin groups. However, the proportion of patients using antiepileptic drugs had increased by > 10% in the SOC alone group at the end of the study. Small increases were also observed in antidepressant and opioid use in the SOC alone group from baseline to the EoS.

## Discussion

In patients with PDPN, capsaicin 8% patch repeat treatment plus SOC over 52 weeks was well tolerated, had no negative functional or neurological effects and raised no new safety concerns compared with SOC therapy.

While the efficacy and safety of a single capsaicin 8% treatment has been previously characterised in patients with PDPN in the double-blind STEP study [16], the open-label PACE study was the first to assess the long-term safety and tolerability of repeated treatment over 52 weeks in PDPN. Capsaicin 8% patch repeat treatment plus SOC was not associated with any deterioration in Norfolk QOL-DN total or subscale scores compared with SOC alone. For the safety endpoints, few differences were observed in results between the 30-min and 60-min capsaicin groups.

Regarding the UENS total score and ‘pinprick sensation’ subscale, and sharp, cold, warm and vibration sensation on standardised neurological tests, no deterioration was observed with capsaicin 8% patch repeat treatment. By EoS, the majority of patients showed no change in sensation category, indicating that the changes observed in Figs. 8a and b and Additional file 6: Figure S4 and Additional file 8: Figure S5 were predominantly in patients who transitioned from ‘absent’ to ‘diminished’ or to ‘normal’ during the study. Taken together, these findings indicate that there was no small fibre mediated sensory loss with capsaicin 8% patch repeat treatment. The sensory testing observations in the subset of patients who received seven consecutive capsaicin treatments demonstrated that regular, repeat treatment with the capsaicin 8% patch was also not associated with deterioration in sensory function.

Alongside the double-blind STEP study, the PACE study formed part of a successful regulatory submission to remove the restriction of treatment with capsaicin 8% patch in patients with diabetes in Europe. The capsaicin 8% patch is now indicated for the treatment of peripheral neuropathic pain in adults either alone or in combination with other medicinal products for pain in Europe [18].

Although the open-label design of this study may be perceived as more representative of capsaicin 8% patch repeat treatment in clinical practice than in a double-blind design, the observed safety evaluations may have been biased by the open-label design. Although the physicians assessing neurological function were blinded to treatment, patients and investigators were unblinded, which may have impacted on the findings. As the primary objective of this open-label study was to assess the safety and tolerability of capsaicin 8% patch repeat treatment, *p*-values were not calculated to supplement the 90% CIs for the between-group differences each month. Furthermore, differences between treatment groups in an open-label study may be caused by the fact that patients know which treatment they are receiving. The LOCF imputation method is a conservative method to estimate the treatment effect. The underlying assumption is that subjects that withdraw have worse efficacy than those that stay in the trial. The LOCF imputation method did use the data of withdrawn subjects and therefore, theoretically, gave worse results in this study than from a non-imputed analysis. As the limitations of the LOCF for missing data methodology are recognised, the results were also analysed using the baseline observation carried forward method, and no differences in the results were observed. Quantitative Sensory Testing (QST) was not performed in this multicenter study and instead, ‘bedside’ sensory testing was used. Although it was not feasible in this multicenter study to provide adequate QST training across all centres, or standardise all assessments in all study centres, potential advantages of QST such as quantification of sensory deficits and allodynia/hyperalgesia, and standardisations of values for several painful sites [22, 23] may have provided greater sensitivity in detecting small variations of thermal or mechanical deficits, and reduced possible variability in testing within and between centres. Other limitations include the impact of concomitant opioid use on the results and that the patient population was 99% Caucasian, and therefore the findings may not be widely applicable to patients of other ethnicity.

## Conclusion

Repeat treatment with the capsaicin 8% patch plus SOC versus SOC alone over 52 weeks in patients with PDPN was well tolerated and consistent with the established safety profile of the capsaicin 8% patch. Capsaicin plus SOC had no negative functional or neurologic effects compared with SOC alone.

## Additional files

Additional file 1: Sensory examination. Details of how sensory examination conducted. (DOCX 111 kb)
Additional file 2: Figure S1. Exposure to capsaicin 8% patch (SAS). Bar chart of number of patients by number of capsaicin treatments. (DOCX 143 kb)
Additional file 3: Figure S2. Maximum number of treatments with capsaicin 8% patch (SAS). Bar chart of number of patients by maximum number of capsaicin treatments. (DOCX 138 kb)
Additional file 4: Table S1. Sensory and reflex testing categories at baseline and end of study (SAS). Table of absolute mean values. (DOCX 35 kb)
Additional file 5: Figure S3. Proportion of patients who reported improved, unchanged, or worsened sensory reflex function by EoS (capsaicin seven treatment cohort). Bar char of proportion of patients by sensory or reflex function. (DOCX 171 kb)
Additional file 6: Figure S4. Change in proportion of patients reporting sensory and reflex testing categories from baseline to EoS (capsaicin seven treatment cohort). Bar chart of change from baseline in patients reporting category by sensory or reflex function. (DOCX 254 kb)
Additional file 7: Table S2. Sensory and reflex testing categories at baseline and end of study (capsaicin seven treatment cohort). Table of absolute mean values. (DOCX 35 kb)
Additional file 8: Figure S5. Change in proportion of patients reporting sensory and reflex testing categories from baseline to EoS (capsaicin seven treatment cohort). Bar chart of change from baseline in patients reporting category by sensory or reflex function. (DOCX 280 kb)
Additional file 9: Table S3. Use of concomitant medications at baseline and at end of study. Table of number of antidepressants, antiepileptic drugs and opioids. (DOCX 33 kb)

## Abbreviations

AE: Adverse event
BPI-DN: Brief pain inventory diabetic neuropathy
CI: Confidence interval
eCRF: Electronic case report form
EMLA: Eutectic mixture of local anaesthetics
EoS: End of study
HbA1c: Glycated haemoglobin of A1c
LOCF: Last observation carried forward
MNSI: Michigan neuropathy screening instrument
N/A: Not applicable
NPRS: Numeric pain rating scale
PDPN: Painful diabetic peripheral neuropathy
PNP: Peripheral neuropathic pain
PRAE: Post randomisation adverse event
QOL: Quality of life
QOL-DN: Quality-of-life questionnaire for diabetic neuropathy
QST: Quantitative sensory testing
SAS: Safety analysis set
SOC: Standard of care
TEAE: Treatment emergent adverse event
UENS: Utah early neuropathy scale
