# Supplementary material for: Capsaicin 8% patch repeat treatment plus standard of care (SOC) versus SOC alone in painful diabetic peripheral neuropathy: a randomised, 52-week, open-label, safety study
Source: BMC Neurol. 2016 Dec 6;16:251. doi: 10.1186/s12883-016-0752-7 (PMC5139122; doi:10.1186/s12883-016-0752-7)

**Appendix 1. Sensory examination**

The objective of the Sensory Examination is to identify clinically relevant deficits in sensory function at baseline and to re-assess the same following investigational treatment with QUTENZA™. Examinations will be performed by neurologists as well as by non-neurologist physician-investigators given study training and findings will be captured using pre-defined scales. Ideally, assessments should be performed by the same person.

**Identification of most painful area(s)**

The QUTENZA treatment area will be mapped at the screening visit and at baseline visit. Treatment area(s) will be identified based on the presence of spontaneous and evoked pain (includes mechanical dynamic allodynia [e.g., pain in response to brush stroke], cold or warm thermal allodynia [e.g., pain in response to touch with a cold or warm object], and/or hyperalgesia [e.g., exaggerated pain in response to pinprick].as determined by a sensory examination performed by the Investigator. Prior to marking the treatment area(s), the subject should be placed in a comfortable recumbent or semi-recumbent position, which will be the same position in which the subject is placed during the patch application period of investigational treatment. The treatment areas will be identified as the areas comprised by the proximal level of painful symptoms around the dorsal, lateral and plantar surfaces of each foot down to the distal extremities, up to a total combined surface area of 1120 cm^2^ for both feet. The boundaries of the areas to be treated should be marked on each foot with a skin marking pen, traced onto tracing paper or plastic transparency.

**Brief Sensory Pain Examination**

The following tests will be performed within the boundaries of the most painful or sensitive area(s). The assessments should be performed at several locations, and only the most intense sensation evoked should be rated.

Details of the brief sensory pain examination are provided below.

***Vibration Sensation***

Demonstration Area:

● Distal interphalangeal joint of either index finger

Test Areas, performed on left and right sides:

● Dorsal surface of great toe (proximal to the nail bed)

Procedure:

Vibration sensation is assessed by striking a 128-Hz tuning fork on the base of the palm hard enough so that a sound is audible, then immediately placing the tip of the tuning fork just proximal to the nail bed and then counting the number of seconds during which the subject feels the vibration. The procedure may be demonstrated on the distal interphalangeal joint of either index finger. Vibration sensation will be assessed on the dorsal surface of the left and right great toes (proximal to the nail beds). Record the findings according to the Vibration Rating Scale, below.

**Vibration Rating Scale**

0 = vibration is not felt (absent)

1 = vibration is felt for < 6 seconds (markedly diminished)

2 = vibration is felt for 6 to 10 seconds (mild loss)

3 = vibration is felt for > 10 seconds (normal)

***Warm Sensation***

Demonstration Area:

● Anterior thigh or upper forearm

Test Areas, performed on left and right sides (see diagram on last page):

1. Ball of foot

2. Midpoint of plantar surface of foot

3. Dorsal surface of great toe (proximal to the nail bed)

4. Midpoint of distal dorsal surface of foot

5. Medial malleolus

Procedure:

Warm sensation will be assessed using a warmed electronic thermometer (Traceable^®^ Surface Dial Thermometer, Cat. No. 4355, CONTROL COMPANY, Friendswood, TX; to be provided to all study centers by Astellas). The thermometer probe will be heated in hot water then removed, dried, and allowed to cool, if needed, until the displayed temperature is 43ºC–44ºC.

*To avoid injury, the thermometer should never exceed 44º C when placed in contact with the subject.*
Ask the subject to keep their eyes closed during the assessment. As a demonstration, apply the distal flat end of the warmed thermometer to the anterior thigh or upper forearm **for 2–3 seconds** and ask the subject to rate what they feel (“not warm, slightly warm, warm or hot, or painfully hot”).
Subsequently, the thermometer should be re-warmed in hot water then removed, dried, and allow it to cool, if needed, until the displayed temperature is 43ºC–44ºC. Apply the distal flat end in sequence to each of the 5 test areas on each foot for **2–3 seconds**. *The thermometer should be re-warmed following each application or as often as needed to maintain a displayed temperature of 43ºC–44ºC prior to a subsequent application*. Ask the subject to rate what they feel during each application of the thermometer (“not warm, slightly warm, warm or hot, or painfully hot”). Capture the subject’s ratings for each test area according to the Warm Rating Scale, below.

If the subject reports the absence of warm sensation at any test area, repeat the test at that location after reheating and verifying the thermometer has a displayed temperature of 43ºC–44ºC. If the absence of warm sensation is verified at a particular test area, indicate this in the eCRF.

**Warm Rating Scale**

0 = warm probe is felt as not warm (absent)

1 = warm probe is felt as slightly warm (diminished)

2 = warm probe is felt as warm or hot (normal)

3 = warm probe is felt as painfully hot (increased)

***Cold Sensation***

Demonstration Area:

● Anterior thigh or upper forearm

Test Areas, performed on left and right sides (see diagram on last page):

1. Ball of foot

2. Midpoint of plantar surface of foot

3. Dorsal surface of great toe (proximal to the nail bed)

4. Midpoint of distal dorsal surface of foot

5. Medial malleolus

Procedure:

Cold sensation will be assessed using the tuning fork dipped in ice water and dried. Ask the subject to keep their eyes closed during the assessment. As a demonstration, apply the distal flat end of the tuning fork to the anterior thigh or upper forearm **for 2-3 seconds** and ask the subject to rate what they feel (“not cold, slightly cold, cold, or painfully cold”). Apply the distal flat end in sequence to each of the 5 test areas on each foot **for 2-3 seconds**. *Make sure to dip the tuning fork in ice water to cool back down following each application*. Ask the subject to rate what they feel during each application of the tuning fork (“not cold, slightly cold, cold, or painfully cold”). Capture the subject’s ratings for each test area according to the Cold Rating Scale, below.

**Cold Rating Scale**

0 = tuning fork is felt as not cold (absent)

1 = tuning fork is felt as slightly cold (diminished)

2 = tuning fork is felt as cold (normal)

3 = tuning fork is felt as painfully cold (increased)

***Sharp Sensation***

Demonstration Area:

● Hand or forearm

Test Areas, performed on left and right sides (see diagram on last page):

1. Ball of foot

2. Midpoint of plantar surface of foot

3. Dorsal surface of great toe (proximal to nail bed)

4. Midpoint of distal dorsal surface of foot

5. Medial malleolus

Procedure:

Ask the subject to keep their eyes closed during the assessment. As a demonstration, gently press the end of a 26 g monofilament to the subject’s hand or arm using a 3 step sequence over **2-3 seconds**: (1) touch the skin, (2) bend the monofilament slightly, and (3) withdraw (see diagram, below).


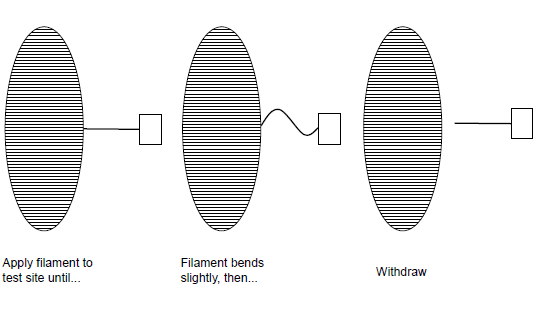


Subsequently, gently press end of monofilament to each of the 5 test areas on each foot in the same manner using a 3 step sequence over **2-3 seconds**: (1) touch the skin, (2) bend the monofilament slightly, and (3) withdraw. Ask the subject to rate what they feel during each application of the monofilament (“not felt, dull, sharp, or painfully sharp”). Capture the subject’s ratings for each test area according to the Sharp Rating Scale, below.

If the subject reports the **absence** of sharp sensation at any test area, repeat the test at that location. If the absence of sharp sensation is verified at a test area, indicate this in the eCRF.

**Sharp Rating Scale**

0 = monofilament is not felt (absent)

1 = monofilament is felt as dull (diminished)

2 = monofilament is felt as sharp (normal)

3 = monofilament is felt as painfully sharp (increased)

***Deep Tendon Reflex***

Test Areas, performed on left and right sides:

● Ankle tendon reflex (Achilles or “ankle jerk”)

Procedure:

● Test ankle reflexes with the subject seated on an examination table, or kneeling backwards on a chair or examination table.

● Percuss the Achilles’ tendon by striking it with the rubber bumper of a long-handled Queen’s Square reflex hammer after bending the foot to be slightly dorsiflexed.

● A normal reflex response results in plantar flexion of the foot.

● Rate the left and right ankle reflexes in terms of muscle tone and velocity of response according to the Reflex Rating Scale, below:

**Reflex Rating Scale**

0 = no response (absent)

1 = hypoactive (diminished)

2 = normal

3 = hyperactive (more brisk than normal)

4 = clonus (very intense)


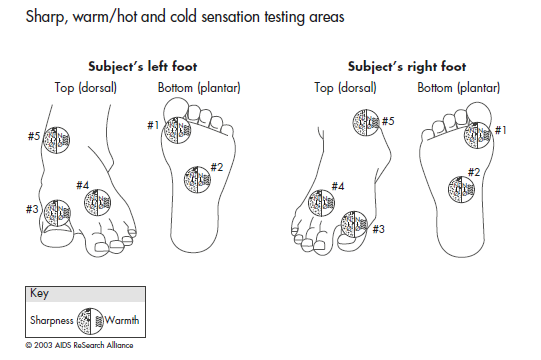

Supplement: Additional file 1: — Sensory examination. Details of how sensory examination conducted. (DOCX 111 kb) [file 12883_2016_752_MOESM1_ESM.docx]
