# Supplementary material for: Capsaicin 8% patch repeat treatment plus standard of care (SOC) versus SOC alone in painful diabetic peripheral neuropathy: a randomised, 52-week, open-label, safety study
Source: BMC Neurol. 2016 Dec 6;16:251. doi: 10.1186/s12883-016-0752-7 (PMC5139122; doi:10.1186/s12883-016-0752-7)
Supplement: Additional file 3: Figure S2. — Maximum number of treatments with capsaicin 8% patch (SAS). Bar chart of number of patients by maximum number of capsaicin treatments. (DOCX 138 kb) [file 12883_2016_752_MOESM3_ESM.docx]

**A2 Fig. Maximum number of treatments with capsaicin 8% patch (SAS).**
