# Supplementary material for: Capsaicin 8% patch repeat treatment plus standard of care (SOC) versus SOC alone in painful diabetic peripheral neuropathy: a randomised, 52-week, open-label, safety study
Source: BMC Neurol. 2016 Dec 6;16:251. doi: 10.1186/s12883-016-0752-7 (PMC5139122; doi:10.1186/s12883-016-0752-7)
Supplement: Additional file 4: Table S1. — Sensory and reflex testing categories at baseline and end of study (SAS). Table of absolute mean values. (DOCX 35 kb) [file 12883_2016_752_MOESM4_ESM.docx]

**A1 Table. Sensory and reflex testing categories at baseline and end of study (SAS)**

| Visit  Absolute mean [SD] | Capsaicin 8% patch (30 min) + SOC  Baseline n=153  EoS n=153 | Capsaicin 8% patch (60 min) + SOC  Baseline n=148  EoS n=155 | SOC  Baseline n=150  – |
| --- | --- | --- | --- |
| Sharp (ball), % at baseline/% at EoS |  |  |  |
| Painful | 2.0/0.7 | 1.4/0.6 | 1.3/0.7 |
| Normal | 11.1/20.9 | 8.8/23.9 | 11.3/13.6 |
| Diminished | 49.0/47.1 | 55.4/47.7 | 60.0/57.1 |
| Absent | 37.9/31.4 | 34.5/27.7 | 27.3/28.6 |
| Sharp (mid plantar), % at baseline/% at EoS |  |  |  |
| Painful | 3.3/2.6 | 3.4/1.3 | 3.3/2.7 |
| Normal | 22.2/28.1 | 21.6/36.1 | 20.7/26.5 |
| Diminished | 47.7/48.4 | 52.7/45.2 | 54.7/56.5 |
| Absent | 26.8/20.9 | 22.3/17.4 | 21.3/14.3 |
| Warm (ball), % at baseline/% at EoS |  |  |  |
| Painful | 1.3/0.0 | 0.0/0.0 | 1.3/0.0 |
| Normal | 7.8/18.3 | 7.4/23.3 | 9.3/16.3 |
| Diminished | 39.9/35.9 | 39.2/45.8 | 35.3/38.8 |
| Absent | 51.0/45.8 | 53.4/31.0 | 54.0/44.9 |
| Warm (mid plantar), % at baseline/% at EoS |  |  |  |
| Painful | 2.0/0.7 | 0.0/0.6 | 1.3/0.0 |
| Normal | 11.1/24.2 | 8.8/28.4 | 14.0/17.7 |
| Diminished | 51.6/44.4 | 50.0/51.6 | 46.0/53.1 |
| Absent | 35.3/30.7 | 41.2/19.4 | 38.7/29.3 |
| Cold (ball), % at baseline/% at EoS |  |  |  |
| Painful | 2.0/0.7 | 3.4/0.6 | 3.3/0.0 |
| Normal | 21.6/32.7 | 23.0/31.6 | 25.3/29.3 |
| Diminished | 40.5/31.4 | 37.2/45.8 | 36.7/36.7 |
| Absent | 35.9/35.3 | 36.5/21.9 | 34.7/34.0 |
| Cold (mid plantar), % at baseline/% at EoS |  |  |  |
| Painful | 3.3/0.7 | 4.1/2.6 | 2.7/0.0 |
| Normal | 29.4/41.2 | 27.7/39.4 | 34.0/34.0 |
| Diminished | 45.8/41.8 | 41.9/45.8 | 40.0/49.0 |
| Absent | 21.6/16.3 | 26.4/12.3 | 23.3/17.0 |
| Vibration (great toe), % at baseline/% at EoS |  |  |  |
| Normal | 3.9/2.6 | 3.4/3.9 | 2.7/4.8 |
| Mild loss | 15.7/24.2 | 12.8/30.3 | 18.7/21.8 |
| Markedly diminished | 41.8/40.5 | 48.0/40.6 | 52.7/49.0 |
| Absent | 38.6/32.7 | 35.8/25.2 | 26.0/24.5 |
| Reflex, % at baseline/% at EoS |  |  |  |
| Hyperactive | 0.0/0.0 | 1.4/0.0 | 0.0/0.0 |
| Normal | 10.5/15.0 | 10.8/12.3 | 9.3/12.9 |
| Diminished | 34.0/30.1 | 35.1/34.8 | 41.3/34.7 |
| Absent | 55.6/54.9 | 52.7/52.9 | 49.3/52.4 |
