# Supplementary material for: Capsaicin 8% patch repeat treatment plus standard of care (SOC) versus SOC alone in painful diabetic peripheral neuropathy: a randomised, 52-week, open-label, safety study
Source: BMC Neurol. 2016 Dec 6;16:251. doi: 10.1186/s12883-016-0752-7 (PMC5139122; doi:10.1186/s12883-016-0752-7)
Supplement: Additional file 5: Figure S3. — Proportion of patients who reported improved, unchanged, or worsened sensory reflex function by EoS (capsaicin seven treatment cohort). Bar char of proportion of patients by sensory or reflex function. (DOCX 171 kb) [file 12883_2016_752_MOESM5_ESM.docx]

**A3 Fig. Proportion of patients who reported improved, unchanged, or worsened sensory or reflex function by EoS (capsaicin seven treatment cohort).** C30 + SOC, capsaicin 8% patch (30 min) + SOC (n=81); C60 + SOC, capsaicin 8% patch (60 min) + SOC (n=76); EoS, end of study; SOC, standard of care (n=143); n is number of patients with non-missing data.
