# Supplementary material for: Capsaicin 8% patch repeat treatment plus standard of care (SOC) versus SOC alone in painful diabetic peripheral neuropathy: a randomised, 52-week, open-label, safety study
Source: BMC Neurol. 2016 Dec 6;16:251. doi: 10.1186/s12883-016-0752-7 (PMC5139122; doi:10.1186/s12883-016-0752-7)
Supplement: Additional file 7: Table S2. — Sensory and reflex testing categories at baseline and end of study (capsaicin seven treatment cohort). Table of absolute mean values. (DOCX 35 kb) [file 12883_2016_752_MOESM7_ESM.docx]

**A2 Table. Sensory and reflex testing categories at baseline and end of study (capsaicin seven treatment cohort)**

| Visit  Absolute mean [SD] | Capsaicin 8% patch (30 min) + SOC  Baseline n=81  EoS n=84 | Capsaicin 8% patch (60 min) + SOC  Baseline n=76  EoS n=83 | SOC  Baseline n=150  EoS n=147 |
| --- | --- | --- | --- |
| Sharp (ball), % at baseline/% at EoS |  |  |  |
| Painful | 0.0/0.0 | 1.3/1.2 | 1.3/0.7 |
| Normal | 11.1/19.0 | 10.5/26.5 | 11.3/13.6 |
| Diminished | 51.9/53.6 | 53.9/45.8 | 60.0/57.1 |
| Absent | 37.0/27.4 | 34.2/26.5 | 27.3/28.6 |
| Sharp (mid plantar), % at baseline/% at EoS |  |  |  |
| Painful | 1.2/1.2 | 3.9/1.2 | 3.3/2.7 |
| Normal | 23.5/29.8 | 18.4/41.0 | 20.7/26.5 |
| Diminished | 50.6/52.4 | 55.3/42.2 | 54.7/56.5 |
| Absent | 24.7/16.7 | 22.4/15.7 | 21.3/14.3 |
| Warm (ball), % at baseline/% at EoS |  |  |  |
| Painful | 1.2/0.0 | 0.0/0.0 | 1.3/0.0 |
| Normal | 4.9/17.9 | 3.9/26.5 | 9.3/16.3 |
| Diminished | 40.7/41.7 | 39.5/47.0 | 35.3/38.8 |
| Absent | 53.1/40.5 | 56.6/26.5 | 54.0/44.9 |
| Warm (mid plantar), % at baseline/% at EoS |  |  |  |
| Painful | 1.2/0.0 | 0.0/0.0 | 1.3/0.0 |
| Normal | 8.6/21.4 | 6.6/32.5 | 14.0/17.7 |
| Diminished | 56.8/48.8 | 51.3/51.8 | 46.0/53.1 |
| Absent | 33.3/29.8 | 42.1/15.7 | 38.7/29.3 |
| Cold (ball), % at baseline/% at EoS |  |  |  |
| Painful | 2.5/0.0 | 3.9/1.2 | 3.3/0.0 |
| Normal | 14.8/32.1 | 18.4/33.7 | 25.3/29.3 |
| Diminished | 49.4/39.3 | 40.8/47.0 | 36.7/36.7 |
| Absent | 33.3/28.6 | 36.8/18.1 | 34.7/34.0 |
| Cold (mid plantar), % at baseline/% at EoS |  |  |  |
| Painful | 2.5/0.0 | 3.9/2.4 | 2.7/0.0 |
| Normal | 25.9/42.9 | 23.7/44.6 | 34.0/34.0 |
| Diminished | 54.3/47.6 | 44.7/39.8 | 40.0/49.0 |
| Absent | 17.3/9.5 | 27.6/13.3 | 23.3/17.0 |
| Vibration (great toe), % at baseline/% at EoS |  |  |  |
| Normal | 2.5/0.0 | 2.6/3.6 | 2.7/4.8 |
| Mild loss | 14.8/27.4 | 14.5/31.3 | 18.7/21.8 |
| Markedly diminished | 44.4/47.6 | 46.1/41.0 | 52.7/49.0 |
| Absent | 38.3/25.0 | 36.8/24.1 | 26.0/24.5 |
| Reflex, % at baseline/% at EoS |  |  |  |
| Hyperactive | 0.0/0.0 | 2.6/0.0 | 0.0/0.0 |
| Normal | 6.2/9.5 | 9.2/9.6 | 9.3/12.9 |
| Diminished | 33.3/32.1 | 32.9/32.5 | 41.3/34.7 |
| Absent | 60.5/58.3 | 55.3/57.8 | 49.3/52.4 |
