# Supplementary material for: Capsaicin 8% patch repeat treatment plus standard of care (SOC) versus SOC alone in painful diabetic peripheral neuropathy: a randomised, 52-week, open-label, safety study
Source: BMC Neurol. 2016 Dec 6;16:251. doi: 10.1186/s12883-016-0752-7 (PMC5139122; doi:10.1186/s12883-016-0752-7)
Supplement: Additional file 8: Figure S5. — Change in proportion of patients reporting sensory and reflex testing categories from baseline to EoS (capsaicin seven treatment cohort). Bar chart of change from baseline in patients reporting category by sensory or reflex function. (DOCX 280 kb) [file 12883_2016_752_MOESM8_ESM.docx]

**A5 Fig. Change in proportion of patients reporting sensory and reflex testing categories from baseline to EoS (capsaicin seven treatment cohort).** C30 + SOC, capsaicin 8% patch (30 min) + SOC (n=84); C60 + SOC, capsaicin 8% patch (60 min) + SOC (n=83); EoS, end of study; SOC, standard of care (n=155).
