# Supplementary material for: Capsaicin 8% patch repeat treatment plus standard of care (SOC) versus SOC alone in painful diabetic peripheral neuropathy: a randomised, 52-week, open-label, safety study
Source: BMC Neurol. 2016 Dec 6;16:251. doi: 10.1186/s12883-016-0752-7 (PMC5139122; doi:10.1186/s12883-016-0752-7)
Supplement: Additional file 9: Table S3. — Use of concomitant medications at baseline and at end of study. Table of number of antidepressants, antiepileptic drugs and opioids. (DOCX 33 kb) [file 12883_2016_752_MOESM9_ESM.docx]

**A3 Table. Use of concomitant medications at baseline and at end of study**

| Visit  n, (%) | Capsaicin 8% patch (30 min) + SOC | Capsaicin 8% patch (60 min) + SOC | SOC |
| --- | --- | --- | --- |
| Baseline | n=156 | n=157 | n=155 |
| Antidepressants | 17 (10.9) | 8 (5.1) | 12 (7.7) |
| Antiepileptic drugs | 44 (28.2) | 49 (31.2) | 50 (32.3) |
| Opioids | 17 (10.9) | 9 (5.7) | 13 (8.4) |
| End of study | n=146 | n=147 | n=146 |
| Antidepressants | 16 (11.0) | 10 (6.8) | 22 (15.1) |
| Antiepileptic drugs | 43 (29.5) | 53 (36.1) | 63 (43.2) |
| Opioids | 16 (11.0) | 12 (8.2) | 17 (11.6) |
